# Continuous-flow left ventricular assist device treatment for arrhythmogenic right ventricular cardiomyopathy complicated by advanced biventricular failure – University of Tokyo experiences

**DOI:** 10.3389/fcvm.2022.1023191

**Published:** 2022-10-05

**Authors:** Minoru Ono

**Affiliations:** Department of Cardiovascular Surgery, The University of Tokyo Hospital, Tokyo, Japan

**Keywords:** arrhythmogenic right ventricular cardiomyopathy, biventricular failure, ventricular assist device, continuous-flow LVAD, heart transplantation

## Abstract

Arrhythmogenic right ventricular cardiomyopathy (ARVC) is an inherited cardiomyocyte disease characterized by intractable ventricular arrhythmia in the majority of affected patients. Some of these patients also manifest right ventricular dysfunction and heart failure symptoms. Fatal ventricular arrhythmia has been the primary cause of death in ARVC patients. However, increased early recognition of ARVC and improvement in arrhythmic risk stratification and treatment have dramatically improved survival. A small proportion of the patients are further complicated by left ventricular impairment at the late phase in addition to right heart failure, for whom only heart transplantation is the last resort. Because of the relative rarity of ARVC with biventricular failure, no consensus or guideline has been reported on how to effectively support these patients with a mechanical circulatory device. Herein, four ARVC patients with biventricular failure were presented who were successfully bridged to heart transplantation after long-term support by isolated continuous-flow LVAD.

## Introduction

Arrhythmogenic right ventricular cardiomyopathy (ARVC) is an inherited cardiomyopathy characterized by fibro-fatty infiltration of the myocardium (1, 2).

Fatal ventricular arrhythmia has been the primary cause of death in ARVC patients. However, increased early recognition of ARVC and improvement in arrhythmic risk stratification and treatment have dramatically improved survival (1). ARVC has a progressive nature and may lead to right ventricular (RV) dysfunction, and in the later phase, and/or in some forms of predominant left ventricular phenotype, left ventricular dysfunction may also occur. Heart transplantation (HTx) is considered for a limited number of ARVC patients who are unresponsive to conventional treatment for ventricular arrhythmia or intractable heart failure. Pathophysiology of heart failure seen in an ARVC patient who is a candidate for HTx represents an advanced stage of biventricular failure. A durable ventricular assist device or total artificial heart (TAH) may be an option to safely await HTx transplantation. There is no consensus regarding what type of mechanical circulatory support device is preferred for this subset of patients. My group previously reported the experiences of treating ARVC patients by cf-LVAD (3). Herein, four successful cases of ARVC along with updated clinical data are presented, which were supported by an isolated continuous-flow left ventricular assist device (cf-LVAD). Our strategies to treat these tough cases by isolated LVAD will also be described.

### Case presentations

Four isolated cf-LVAD cases for a bridge to transplantation in patients with ARVC were presented. There was no case of TAH or RVAD to support an ARVC patient. Table 1 shows the patients’ demographics. Perioperative and follow-up data are presented in Table 2. In all cases, cf-LVAD implantation was decided after careful discussion with the heart team. The possibility of additional RVAD was always taken into consideration. Our strategy to support a cf-LVAD patient complicating RV dysfunction is to routinely use inhaled nitric oxide (iNO) before weaning cardiopulmonary bypass (CPB). iNO was replaced by an oral phosphodiesterase-5 inhibitor (PDE-5I) in the ICU to reduce RV afterload. Routine and repeated echocardiography and right heart catheterization, if necessary, are key examinations to make sure that a patient is on optimal (or acceptable) LVAD support. All four ARVC patients reached to HTx, although three patients had VAD-related complications during a follow-up.

**TABLE 1:** Patient demographics.

	Case 1	Case 2	Case 3	Case 4
ARVC diagnosis	Before LVAD	Before LVAD	Before LVAD	After HTx
Age at LVAD	32	57	35	52
Gender	Male	Male	Male	Male
Height	168	168	160	171
Weight	56	51	46	58
Body surface area (m2)	1.64	1.57	1.45	1.68
Body mass index (kg/m2)	19.8	18.1	18.0	19.8
Antiarrhythmics (mg/day)	Sotalol (80)	Amiodarone (100) Digoxin (0.125)	Amiodarone (100)	Amiodarone (100)
Beta-blocker (mg/day)	Carvedilol (20)	Carvedilol (10)	Carvedilol (1.25)	Carvedilol (20)
ACEI or ARB (mg/day)	Enalapril (2.5)	None	None	Enalapril (10)
Diuretics (mg/day)	Tolvaptan (3.75)	Furosemide (40) Tolvaptan (7.5)	Furosemide (10)	Azosemide (30)
MRA (mg/day)	Spironolactone (25)	None	Spironolactone (12.5)	Eplerenone (25)
PDE-3 inhibitor (mg/day)	None	Pimobendan (2.5)	Pimobendan (5.0)	Pimobendan (1.25)
Inotropic agents (μg/kg/min)	DOA (2), DOB (3)	DOB (4)	DOB (3)	DOB (2)
BNP (pg/mL)	2158	1094	593	520

ARVC, arrhythmogenic right ventricular cardiomyopathy; LVAD, left ventricular assist device; ACEI, angiotensin-converting enzyme inhibitor; ARB, angiotensin receptor blocker; MRA, mineral corticoid receptor antagonist; PDE, phosphodiesterase; BNP, brain natriuretic peptide; HTx, heart transplantation; DOA, dopamine; DOB, dobutamine.

**TABLE 2:** Perioperative and follow-up data.

	Case 1	Case 2	Case 3	Case 4
Age at LVAD	32	57	35	52
Cf-LVAD	DuraHeart	EVAHEART	Jarvik 2000	EVAHEART
Concomitant procedures	ATL pericardial patch augmentation, TAP	TVR	TVR	ATL pericardial patch augmentation, edge-to-edge repair, TAP
Pump time (min)	160	229	158	191
Aortic cross-clamp time (min)	0	0	0	0
Nitric oxide inhalation	Yes	Yes	Yes	Yes
Mechanical ventilation (hours)	65	111	184	68
Postop inotropic support (days)	8	21	17	80
Postop ICU stay (days)	7	12	18	11
Postop PDE-5 inhibitor	yes	yes	yes	yes
Index hospitalization (days)	68	78	91	88
Pump exchange	Twice	None	None	None
Readmission for cardiac event	Yes (VA, HF)	None	Yes (HF)	None
Support duration (days)	929	1607	1663	1745
Outcome	HTx	HTx	HTx	HTx

Cf-LVAD, continuous-flow left ventricular assist device; ICU, intensive care unit; PDE, phosphodiesterase; ATL, anterior tricuspid leaflet; TAP, tricuspid annuloplasty.

### Case 1

**FIGURE 1:**
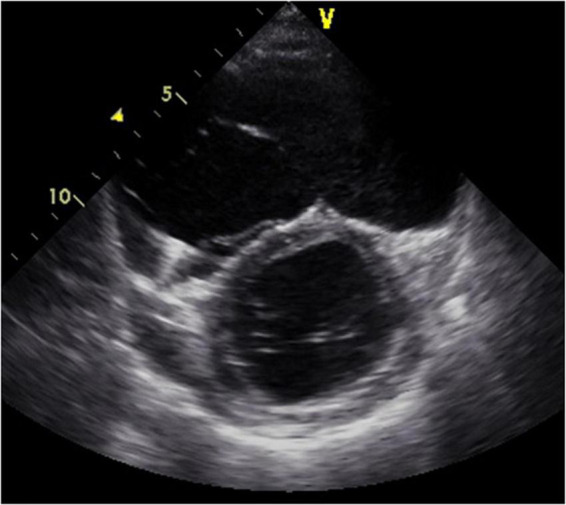
Short-axis view at end-diastolic phase of transthoracic echocardiography in Case 1. Note that the right ventricle is much larger than the left ventricle.

**FIGURE 2:**
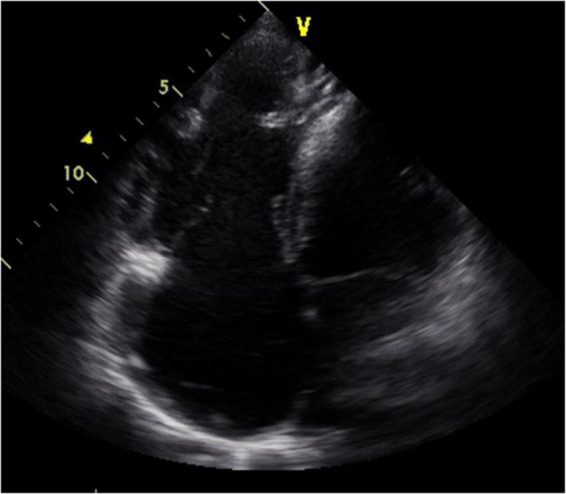
Four-chamber view at end-systolic phase of transthoracic echocardiography in Case 1. Note that the tricuspid valve does not close at all, and the right atrium is hugely dilated.

A 32-year-old man suffering from advanced heart failure due to ARVC was transferred to our hospital for a close work-up for HTx. He was diagnosed with ARVC 11 years ago and implanted with a cardiac resynchronization therapy defibrillator (CRT-D) 1 year ago. Echocardiography demonstrated a left ventricular ejection fraction (LVEF) of 16% and a left ventricular end-diastolic diameter (LVDd) of 55 mm. The right ventricle was hugely enlarged with markedly reduced RV function with RV fractional area change (RVFAC) of 14% and tricuspid annular plane systolic excursion (TAPSE) of 4 mm, accompanying severe tricuspid regurgitation (TR) with advanced leaflet tethering (Table 3 and Figures 1, 2). Cardiac catheterization revealed RV stroke work index (RVSWI) was 0 g m/m^2^/beat and cardiac index (CI) was as low as 1.5 L/min/m^2^ on dobutamine 5 μg/kg/m^2^ (Table 4). After in-depth discussion, the heart team reached the conclusion to implant a cf-LVAD, considering the long waiting time in Japan. He underwent a DuraHeart (Terumo Heart, Ann Arbor, MI, USA) implantation and tricuspid annuloplasty (TAP) with autologous pericardial patch augmentation of the anterior tricuspid leaflet (ATL). Histology of the resected left ventricular (LV) apex showed myocardial tissue with interstitial fibrosis and fatty infiltration. Inhalation of NO was started before coming off CPB. Meticulous weaning with a moderate amount of dobutamine and milrinone enabled separation from CPB without RVAD by gradual titration of cf-LVAD speed. PDE-5I was administered via a nasogastric tube to reduce an afterload of RV in the ICU, and iNO inhalation could be terminated postoperative day 3 (POD3). A ramp test performed 1 month after cf-LVAD implantation showed acceptable hemodynamics with a CI of 2.41 L/min/m^2^ (Table 4). He was discharged home 68 days after cf-LVAD implantation. He had multiple readmissions. He suffered from atrial fibrillation (Af) requiring cardioversion 255 POD. He had two documented episodes of ventricular tachycardia (VT) requiring readmission at 520 and 648 PODs. He was also readmitted 548 POD for magnetic levitation system failure and due to heart failure 801 POD. Serial follow-up echocardiography showed gradual worsening of TR and aortic insufficiency (AI) (Table 3). He was readmitted 885 POD for heart failure due to severe AI and severe TR for which repeated catheter examinations were conducted (Table 4). He underwent aortic valve repair (Park’s stitch) and tricuspid valve replacement (TVR) using a bioprosthetic valve. Three days after the valvular surgery, the pump exchange (DuraHeart to DuraHeart) was performed for pump thrombosis. Pump thrombosis recurred 10 days after valve surgery, and he underwent a conversion surgery to a Jarvik 2000 (Jarvik Heart, Inc., New York, NY, USA). After the second pump exchange, his hemodynamic status became stabilized. Finally, he underwent a successful HTx 929 days after DuraHeart insertion. The pathological examination confirmed the typical findings of ARVC. He did very well 5 years after HTx.

**TABLE 3:** Trend of echocardiographic data in Case 1.

	Before LVAD	1 month	1 year	2 year	2 year 5 months
LVDd (mm)	55	57	54	56	62
LVEF (%)	16	4	8	4	4
RVFAC (%)	14	13	13	6	15
TAPSE (mm)	4	5	7	7	7
Aortic insufficiency	None	Trivial	Mild	Moderate	Severe
Tricuspid regurgitation	Severe	Severe	Severe	Severe	Severe

LVDd, left ventricular end-diastolic dimension; LVEF, left ventricular ejection fraction; RVFAC, right ventricular fractional area contraction; TAPSE, tricuspid annular plane systolic excursion; LVAD, left ventricular assist device.

**TABLE 4:** Trend of right heart catheter study in Case 1.

	Before LVAD	1 month	2 year	2 year 2 months
DuraHeart speed (rpm)	–	1650	1700	1900
RAP (mmHg)	17/21/16	14/14/12	28/30/24	15/16/14
RVP (mmHg)	22/edp17	14/edp10	30/edp27	16/edp12
PAP (mmHg)	21/13/16	14/10/13	30/22/24	16/11/14
PCWP (mmHg)	17/14/14	8/8/8	20/20/16	8/9/8
CO/CI (by Fick)	2.54/1.52	4.11/2.41	4.06/2.42	3.35/1.97

LVAD, left ventricular assist device.

### Case 2

**FIGURE 3:**
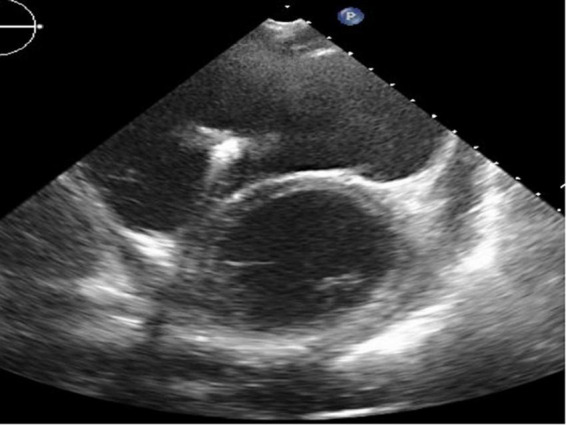
Short-axis view at end-diastolic phase of transthoracic echocardiography in Case 2. Note that the right ventricle is much larger than the left ventricle.

**FIGURE 4:**
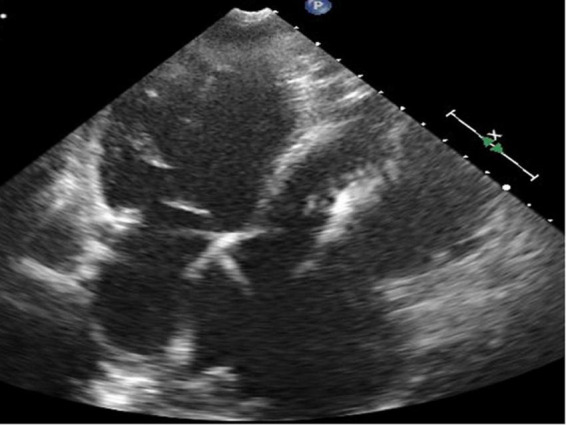
Four-chamber view at end-systolic phase of transthoracic echocardiography in Case 2. Note that the tricuspid valve does not close at all, and the right atrium is hugely dilated.

A 57-year-old man who had a history of ARVC for 19 years was transferred to our hospital for listing for HTx. He had multiple episodes of sustained ventricular tachycardia (SVT) and underwent frequent catheter ablation therapies, resulting in CRT-D insertion 5 years ago. Echocardiography revealed dilated LVDd (60 mm) and severely depressed LVEF (10%), and huge RV with markedly low RV systolic function of RVFAC 10% (Table 5 and Figures 3, 4). As part of his work-up, he had cardiac catheterization which showed decreased CI of 1.21 L/min/m^2^, substantially no RV forward work (RVSWI 0 g m/m^2^/beat), and elevation of pulmonary vascular resistance of 4.21 HRU. Clinical deterioration prompted an intra-aortic balloon pump (IABP) insertion in addition to inotropic support. He was listed for HTx. The heart team discussion reached a consensus to support the patient by cf-LVAD. EVAHEART (Sun Medical Technology Research Corporation, Nagano, Japan) was chosen considering the device characteristics of a very flat HQ curve. TR was treated by TVR. Histology of the resected LV apex showed myocardial tissue with interstitial fibrosis and fatty infiltration. iNO was started at the time of CPB weaning, and again, the patient was successfully switched to isolated cf-LVAD support. iNO was continued for 5 days during which PDE-5I was administered. Ramp test 1 month after cf-LVAD insertion showed sufficient CI of 2.73 L/min/m^2^ with still high mean right atrial pressure of 16 mmHg. He was discharged home on POD 78. He had no episode of cardiovascular events nor device-related complications during follow-up, and he never experienced readmission for any reason. The patient was finally transplanted after 1,607 day-long support. Post-HTx course was uneventful. The pathological examination confirmed the typical findings of ARVC.

**TABLE 5:** Trend of echocardiographic data in Case 2.

	Before LVAD	1 month	1 year	2 years	3 years	4 years
LVDd (mm)	58	60	66	67	57	66
LVEF (%)	10	18	5	13	25	20
RVFAC (%)	10	8	7	20	8	3
TAPSE (mm)	–	6	–	–	4	–
Aortic insufficiency	None	Mild	Mild	Mild-moderate	Moderate	Moderate
Tricuspid regurgitation	Moderate	Severe	Severe	Severe	Severe	Severe

LVDd, left ventricular end-diastolic dimension; LVEF, left ventricular ejection fraction; RVFAC, right ventricular fractional area contraction; TAPSE, tricuspid annular plane systolic excursion; LVAD, left ventricular assist device.

### Case 3

**FIGURE 5:**
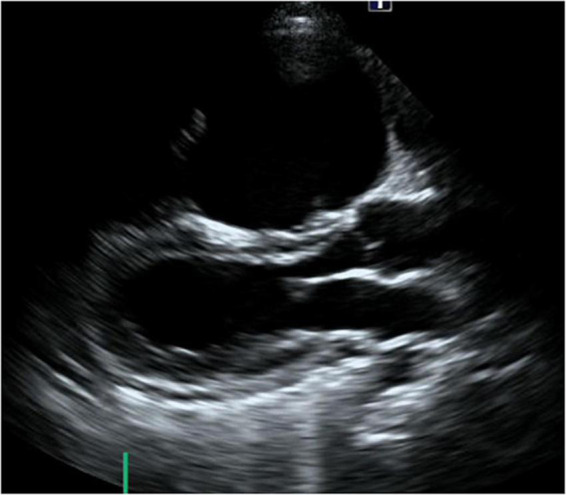
Long-axis view at end-diastolic phase of transthoracic echocardiography in Case 3. Note that the right ventricle is much larger than the left ventricle, and the left ventricle is very small (end-diastolic dimension 31 mm).

**FIGURE 6:**
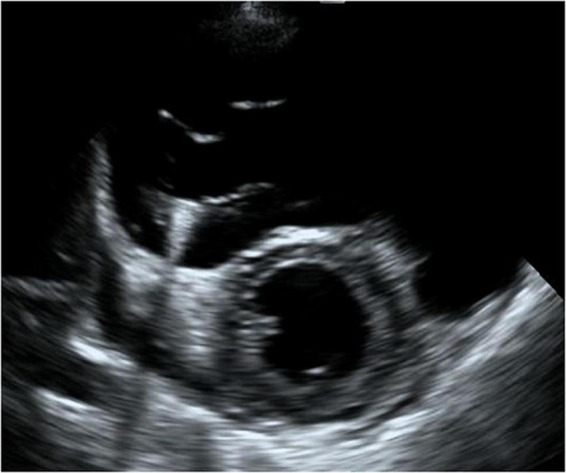
Short-axis view at end-diastolic phase of transthoracic echocardiography in Case 3. Note that the right ventricle is much larger than the left ventricle, and the left ventricle is very small.

A 35-year-old man with a history of ARVC for 17 years was referred to our hospital for an evaluation of heart failure and possible listing for HTx. An implantable cardioverter defibrillator (ICD) was inserted 6 years ago. Echocardiography demonstrated reduced LVEF (15%) and very small LV (LVDd 31 mm). His RV was remarkably enlarged with reduced RV systolic function (RVFAC 26%), and TR was graded as severe (Table 6, Figures 5, 6, and Supplementary Movies 1, 2). Cardiac catheterization revealed CI was very low as 1.08 L/min/m^2^ on dobutamine 5 μg/kg/min and no effective RV stroke work as shown by RVSWI 0 g m/m^2^/beat (Table 7). Because of clinical deterioration, an IABP was inserted. After listing him to HTx, the heart team had a heated discussion about whether cf-LVAD implantation should be pursued. Considering that there was no other treatment left, cf-LVAD implantation was determined. Considering his small body size and body surface area of 1.45 m^2^, he underwent a Jarvik 2000 insertion and TVR. Histological findings showed fibro-fatty replacement of the myocardium. iNO was started during the operation, and it was used for 5 days in the ICU. iNO inhalation was successfully replaced by PDE-5I treatment. Although he suffered from brain infarction on 2 POD, he recovered well and was discharged home 91 days after LVAD insertion. A ramp test was performed 1 month after LVAD insertion. CI was not high enough (2.26 L/min/m^2^) but much improved, and RVSWI remained zero (Table 7). He was readmitted on two occasions on 175 and 630 PODs due to right heart failure. Readjustment of medication (an increase of diuretics and reduction of Carvedilol) and volume adjustment could relieve his symptoms. He successfully underwent HTx on 1,663 POD. The pathological examination confirmed the typical findings of ARVC. He was doing well at the outpatient clinic.

**TABLE 6:** Trend of echocardiographic data in Case 3.

	Before LVAD	1 month	1 year	2 years	3 years	4 years	4 year 6 months
LVDd (mm)	31	31	36	42	40	41	41
LVEF (%)	15	29	22	26	22	17	21
RVFAC (%)	26	8	6	7	13	5	4
TAPSE (mm)	4	0	–	3	4	–	3
Aortic insufficiency	None	Mild	Mild-moderate	Mild-moderate	Mild-moderate	Moderate	Moderate-severe
Tricuspid regurgitation	Severe	Severe	Severe	Severe	Severe	Severe	Severe

LVDd, left ventricular end-diastolic dimension; LVEF, left ventricular ejection fraction; RVFAC, right ventricular fractional area contraction; TAPSE, tricuspid annular plane systolic excursion; LVAD, left ventricular assist device.

**TABLE 7:** Trend of right heart catheter study in Case 3.

	Before LVAD	1 month	2 months	6 months	1 year 8 months
Jarvik 2000 speed (rpm)	–	8000	9000	9000	9000
RAP (mmHg)	13/14/11	16/19/16	18/21/18	17/18/17	(15)
RVP (mmHg)	13/edp9	24/edp10	23/edp12	15/edp11	13
PAP (mmHg)	15/13/11	19/12/18	26/14/19	20/15/18	(14)
PCWP (mmHg)	4/5/4	19/18/17	10/13/12	6/8/8	(8)
CO/CI (by Fick)	1.60/1.08	2.61/1.75	2.75/1.84	3.49/2.32	3.72/2.49

LVAD, left ventricular assist device.

### Case 4

**FIGURE 7:**
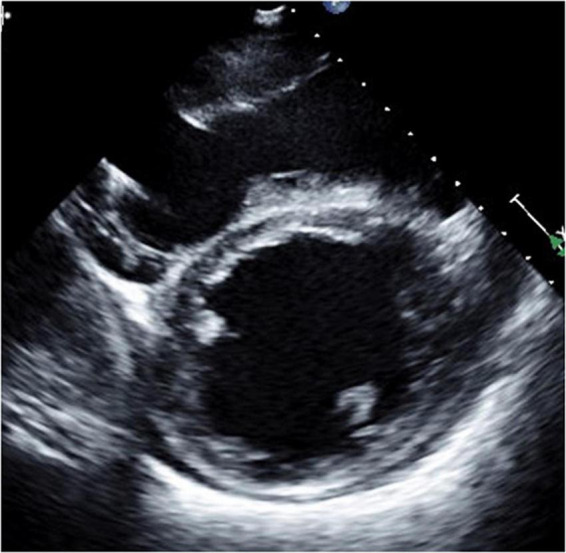
Short-axis view at end-diastolic phase of transthoracic echocardiography in Case 4.

**FIGURE 8:**
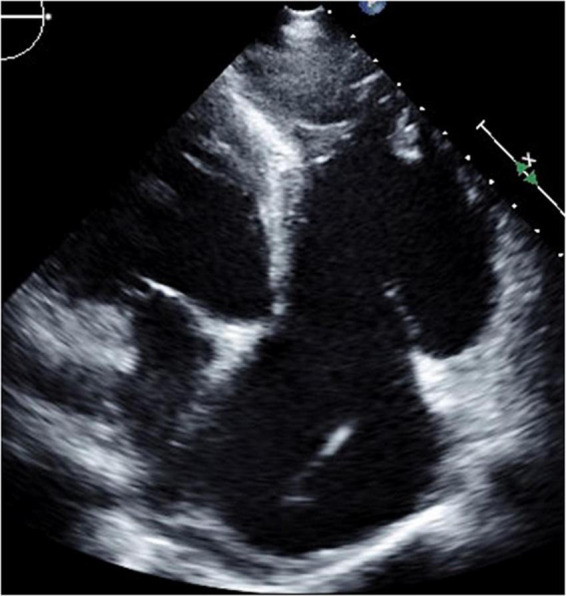
Four-chamber view at end-systolic phase of transthoracic echocardiography in Case 4. Note that the tricuspid valve does not close at all, and the right atrium is hugely dilated.

A 52-year-old man with multiple episodes of SVT and subsequent several catheter ablation therapies was referred to our hospital for a tentative diagnosis of idiopathic dilated cardiomyopathy (DCM) due to inotropic-dependent heart failure accompanying renal and hepatic dysfunction. He was implanted with ICD 3 years ago. Echocardiography showed remarkable biventricular enlargement with severely depressed LV function (LVDd 69 mm and LVEF 3%) and poor RV function (RVFAC 19%) with severe TR (Figures 7, 8 and Table 8). The right heart catheterization test showed a low CI of 1.85 L/min/m^2^ with RVSWI below zero (mRAP 15 mmHg and mPAP 14 mmHg). He was listed for HTx and underwent EVAHEART implantation considering the patient’s very poor RV function. TR was treated by TAP with ATL patch augmentation and a couple of edge-to-edge repairs. iNO was started during weaning from CPB, and the patient was successfully supported by isolated cf-LVAD. iNO was used until 2 POD, and PDE-5I was administered. A ramp study performed 1 month after cf-LVAD insertion showed a little improved CI of 2.24 L/min/m^2^ with unchanged RVSWI. It took more than 2 months to stop dobutamine infusion due to RV failure, and he was discharged home at 97 POD. He was readmitted twice due to ischemic stroke events, resulting in right hemiparesis and mild dysarthria. He reached HTx 1,745 days after EVAHEART implantation. Post-HTx course was uneventful. Histological examination of the resected heart revealed a wide range of fibro-fatty replacements mainly in the RV. The final pathological diagnosis was ARVC.

**TABLE 8:** Trend of echocardiographic data in Case 4.

	Before LVAD	1 month	1 year	2 year	3 year	4 year	4 year 6 months
LVDd (mm)	69	67	73	62	61	69	64
LVEF (%)	5	10	9	9	4	13	5
RVFAC (%)	19	14	15	13	17	18	13
TAPSE (mm)	–	4	3	–	–	5	7
Aortic insufficiency	None	Trivial	Trivial	Trivial	Mild	Mild	Mild
Tricuspid regurgitation	Severe	Moderate	Severe	Mild-moderate	Severe	Severe	Severe

LVDd, left ventricular end-diastolic dimension; LVEF, left ventricular ejection fraction; RVFAC, right ventricular fractional area contraction; TAPSE, tricuspid annular plane systolic excursion; LVAD, left ventricular assist device.

## Discussion

Arrhythmogenic right ventricular cardiomyopathy is an inherited cardiomyocyte disease characterized by fibro-fatty infiltration of the myocardium (1, 2). The right ventricle is predominantly affected, but in the later disease process, the left ventricle may be also involved in at least 50% of cases, with some forms showing a predominant left ventricular phenotype (4). The first clinical presentation of ARVC is ventricular arrhythmia with various levels of right ventricular dysfunction. Diagnostic criteria based on structural abnormalities, fatty or fibro-fatty replacement of the RV myocardium, electrocardiographic changes, arrhythmias of RV origin, and familial disease were updated as the 2010 Task Force Criteria (2). Fatal ventricular arrhythmia has been the primary cause of death in these patients. However, increased early recognition of ARVC and improvement in arrhythmic risk stratification and treatment with anti-arrhythmic medication, catheter ablation, and ICD implantation have dramatically improved survival (1). ARVC has a progressive nature and may lead to RV dysfunction, and in the later phase, and/or in some forms of predominant left ventricular phenotype, left ventricular dysfunction may also occur (5). HTx is considered for a few ARVC patients who are unresponsive to conventional treatment for ventricular arrhythmia or intractable heart failure (6). Previous studies have estimated the incidence of heart failure in patients with ARVC to be 5% and the necessity of HTx to be 2% (7, 8). HTx can be utilized in this population for end-stage HF or refractory ventricular arrhythmias, with outcomes reported primarily in case reports or single institution case series (9–12). Gilljam et al. reported 31 HTx cases performed between 1988 and 2014 enrolled in the Nordic ARVC Registry, of whom 18 had a biventricular failure, 9 had a right ventricular failure, and 1 had left ventricular failure (13). But, there was no patient who was implanted with a mechanical circulatory support device in this cohort. Guiliano et al. reported 252 HTx wait-listed patients extracted from the UNOS database between 1994 and 2020 (14). They comprised only 0.4% of the entire HTx listed patients, but the percentage of all HTx performed for the primary indication of ARVC increased from 0.04% in 1994 to 0.73% in 2019. Their median age was 48 [inter-quartile range (IQR) 30–58] years, and 65.1% (*n* = 164) were men. The majority (85.3%, *n* = 215) had an ICD implanted. One-hundred-eighty-nine patients (75%) underwent HTx. Six patients (2.4%) had VADs on listing: one with LVAD, two with a TAH, and three with BiVAD. By the time of transplantation, a total of 15 patients (7.9%) had VADs: six with LVADs, two with RVADs, four with TAHs, and three with BiVAD (14).

In ARVC, ventricular dysfunction starts from the right ventricle, and in very few cases, severe left ventricular dysfunction manifested in the later phase of disease progression. This implicates that almost all ARVC patients who present left ventricular failure also have right ventricular failure. The clinical course of four cases was summarized, in which severe biventricular failure was present at the time of HTx listing. In the report by Guiliano et al., seven patients required biventricular support either by TAH or BiVAD, and only six patients were supported by isolated LVAD (10). My group protocolized intraoperative management of cf- LVAD support after experiencing the first case. NO inhalation at 20 ppm, dobutamine 5 μg/kg/min, and milrinone 0.5 μg/kg/min infusion were started well before CPB weaning. An angle of inflow cannula is essential to avoid sucking, which may lead to insufficient blood flow and ventricular arrhythmias by irritation of the left ventricle. If an inflow angle is not perfect under transesophageal echo, correction of the inflow direction needs to be undertaken. Our practice is to take sufficient time for deairing from the left ventricle and atrium through both the native aortic valve and the LVAD. Entry of even a small amount of air into the right coronary artery could worsen right ventricular function. In order to see if a patient can tolerate isolated cf-LVAD support, close observation by echocardiography and the hemodynamic parameters is mandatory. Because the stroke volume of RV is lower than other diseases, paced heart rate is usually set at 100 bpm. Sequential pacing is preferred to make the best of atrial kick to send the blood to the pulmonary artery (15). Close hemodynamic observation is continuously undertaken during a half flow of CPB, and the cf-LVAD drive starts at a low speed. CVP is increased to 12–15 mmHg, and CPB support is reduced to a minimal level. Cf-LVAD speed is titrated up to the least acceptable number. In this scenario, an estimated LVAD support at the time of CPB termination was usually 1.8–2.0 L/min/m^2^. Protamine is given very slowly, because its infusion may cause reactive pulmonary artery hypertension and/or systemic hypotension by vasodilation. In the ICU, repeated transthoracic echocardiographic evaluations are mandatory mainly to observe LV size. LVDd > 45 mm is the least acceptable size except for Case 3. If LVDd is larger than 50 mm and the estimated LVAD flow index is less than 2.2 L/min/m^2^, minimal up-titration of pump speed (i.e., 50 – 100 rpm) is considered. iNO is replaced by PDE-5I. The efficacy of PDE-5I for right heart failure in the setting of no pulmonary hypertension is a matter of debate. But my group has experienced a good response in many other cases in which PDE-5I was effective to protect the impaired RV after cf-LVAD insertion. The patient’s hemodynamics were routinely evaluated by simultaneous right heart catheterization and echocardiography 1 month after cf-LVAD implantation to see if cf-LVAD support is optimal or readjustment is required. In Cases 1 and 3, right heart catheterization was repeated because the patient’s symptoms and volume status were not just right even after pump speed adjustment under transthoracic echocardiographic guidance. In Case 1, pump speed was up-titrated based on the catheterization test results.

The ARVC patients who require HTx due to heart failure usually have severe TR, which is caused by severe dilatation of RV as seen in the cases. Severe TR was treated either by TAP with ATL patch augmentation (a large patch is mandatory) or TVR using bioprosthesis. As shown in the trend of echocardiographic results, even TVR could not eliminate regurgitation, and the TR grade continued to be severe during a follow-up. Close observation of leaflet motion of the replaced bioprosthesis showed that only minimal leaflet closure motion was seen at the systolic phase, probably because RV contractile power in these cases was not strong enough to push back these leaflets. Another common finding in our cases was that AI progressed significantly during a follow-up despite no AI before cf-LVAD insertion. This deterioration of aortic valve competency is thought to be due to poor LV filling, which may lead to low LV pressure throughout the cardiac cycle. The continuous lower pressure of LV than the aorta and constant closure of the aortic valve may be the reason for this late complication. Aortic valve repair was performed along with TVR in Case 1, but this intervention triggered a subsequent stormy postoperative course. Late aortic valve surgery in an LVAD patient who has biventricular failure should be decided by meticulous evaluation and team discussion. Prophylactic aortic valve surgery at the time of cf-LVAD insertion may be an option. To avoid late valvular issues of both severe TR and severe AI, TAH might be a better option. BiVAD as an initial surgery would be complicated by the same valve issues as far as tricuspid and aortic valves remain in position. If a high allocation priority is set for TAH as in the US, TAH may be a viable option for the treatment of ARVC with biventricular dysfunction. There is no data yet on what mechanical circulatory support configuration is better than the others in the setting of ARVC. A large-scale clinical study is necessary to find out the best mechanical circulatory support for ARVC, although this may be practical.

## Conclusion

Four successful cases in which isolated cf-LVAD support to HTx was given are presented. Our strategies to treat and manage this difficult pathophysiology are also described. TAH may be the best option for mechanical circulatory support where an appropriate allocation policy of HTx for this specific disease is effective to make a waiting time within a reasonable length. A large-scale clinical study is necessary to find out the best mechanical circulatory support, particularly for long support exceeding a year.

## Data availability statement

The original contributions presented in this study are included in the article/Supplementary material, further inquiries can be directed to the corresponding author.

## Ethics statement

The studies involving human participants were reviewed and approved by The University of Tokyo 3031-(4). The patients/participants provided their written informed consent to participate in this study.

## Author contributions

MO collected and analyzed the clinical information including echocardiography and catheterization, wrote the whole manuscript.

## Supplementary material

Supplementary Movie 1 Long-axis view of transthoracic echocardiography in Case 3. Note that the right ventricle is much larger than the left ventricle and the left ventricle is very small (end-diastolic dimension 31 mm).

Supplementary Movie 2 Four-chamber view of transthoracic echocardiography in Case 3. Note that the tricuspid valve does not close at all and tricuspid regurgitation is severe.
